# Mutual pathways between peer and own e-cigarette use among youth in the United States: a cross-lagged model

**DOI:** 10.1186/s12889-023-16470-5

**Published:** 2023-08-24

**Authors:** Hui G. Cheng, Pavel N. Lizhnyak, Nadja Richter

**Affiliations:** Altria Client Services LLC, 601 E. Jackson, Richmond, VA 23219 USA

**Keywords:** E-Cigarettes, Adolescents, United States, Peer influence

## Abstract

**Background:**

Electronic cigarettes (e-cigarettes) have become the most common tobacco product used among adolescents in the United States (US). Prior research has shown that peer e-cigarette use was associated with increased risk of own e-cigarette use. Nonetheless, there is little empirical evidence on the directionality of these associations—if peer use predicts own use (peer influence) or if own use predicts peer use (peer selection).

**Methods:**

We estimated the association between peer and own e-cigarette use among US adolescents 12–17 years of age. We used the cross-lagged model to investigate the mutual relationship between peer and own e-cigarette use over time using data from a population-based longitudinal study, Population Assessment of Tobacco and Health. Stratified analyses were conducted by sex and age subgroups.

**Results:**

Results from a cross-lagged model showed a statistically significant predicting path leading from peer use at the prior time point to own use at the following time point, but not vice versa.

**Conclusions:**

We found strong relationships between peer e-cigarette use and own e-cigarette use at within-individual levels. Peer influence paths were more robust than peer selection paths for e-cigarette use. Incorporating peers into prevention and intervention programs may help enhance these strategies.

**Supplementary Information:**

The online version contains supplementary material available at 10.1186/s12889-023-16470-5.

## Background

Electronic cigarettes (e-cigarettes) have become the most commonly used tobacco product among adolescents and young adults in the United States (US). According to the National Youth Tobacco Survey (NYTS), a nationally representative school survey of middle and high school students, 14.1% of high school students and 3.3% of middle school students reported e-cigarette use during the 30 days prior to the survey in 2022 [1].

Peer use has been identified as one of the most prominent variables associated with tobacco use among adolescents [2–9], and friends’ use was commonly cited as a top reason to use tobacco, including e-cigarettes [10]. As e-cigarettes gained popularity, a few studies documented that friends’ approval and use of e-cigarettes were strongly associated with own e-cigarette use among a range of variables studied [3, 4, 7]. A more recent study among a nationally representative sample of 12^th^ graders found that social competence and positive peer influence, including the perceived prevalence of substance use among peers, were protective factors against both nicotine and marijuana vaping [11]. Other work has found that adolescents may emulate their peers’ behaviors and may initiate the use of specific e-cigarette brands or flavors based on peer use [7, 12]. Taken together, the results of these studies suggested that peers may play an important role in youth use of e-cigarettes.

Some studies have found sex- and age-variations in peer and own tobacco use relationships. For example, Liao and colleagues found stronger peer-and-own-cigarette-smoking relationships among late-adolescent girls compared to boys [13]. In another study, Duan and colleagues found that 12–15-year-old girls with support from classmates smoked cigarettes on more days whereas no such relationship was found in boys [6]. With respect to e-cigarette use, a recent study among 15–20-year-olds found that, although having friends who used e-cigarettes was associated with current e-cigarette use among those who were 16 years of age and older for both boys and girls, the strength of association tended to decrease among females and increase among males with age [14]. These studies suggest potential variations by sex and age. In addition, given that e-cigarette use and access vary by age groups and between boys and girls [15], it is of interest to explore potential variations.

While previous studies have consistently found evidence for social homophily in youth tobacco use and have highlighted the significance of peers in youth e-cigarette use [11, 14], there are still several areas that require further investigation to enhance our understanding of the role of peer e-cigarette use and inform prevention and intervention strategies.

In a cross-sectional setting, the directionality between peer and own e-cigarette use is less clear. Two underlying pathways can contribute to the observed homophily in youth tobacco use: peer influence (e.g., youth using tobacco to be more like their tobacco-using friends) and peer selection (e.g., tobacco-using youth seeking out friends who also use tobacco). Previous studies on youth cigarette smoking have found evidence for both peer influence and peer selection [16–19]. As youth cigarette smoking continues to decline and e-cigarettes have become the most common tobacco product used [15], it is necessary to systematically investigate the role of peer use over time, as peer use remains one of the most important factors in youth tobacco use. By understanding how peer influence and peer selection operate in the context of e-cigarette use, we can better inform prevention and intervention strategies.

Against this background, we aimed to assess the mutual relationships between peer and own e-cigarette use among adolescents. Using data from a nationally representative longitudinal study of 12- to 17-year-olds in the US we sought to assess the mutual relationships of peer e-cigarette use and own e-cigarette use among youth. That is, whether peer influence or peer selection is more prominent in youth e-cigarette use. We further stratify our analysis by sex and age to explore potential subgroup variations.

## Methods

### Study population and sample

In this study, the population of interest was US non-institutionalized civilian adolescents 12–17 years of age. Data were from the longitudinal Population Assessment of Tobacco and Health (PATH) study and include data from 5 timepoints: wave 2 (Oct. 2014–Oct. 2015), wave 3 (Oct. 2015–Oct. 2016), wave 4 (Dec. 2016–Jan. 2018), wave 4.5 (Dec. 2017-Dec. 2018), and wave 5 (Dec. 2018-Nov. 2019) surveys. Because peer e-cigarette use was not assessed in the first PATH wave, we did not include data from wave 1. PATH employed a multi-stage sampling method to draw nationally representative samples after Institutional-Review-Board-approved parent consent and youth assent [20]. In contrast to school surveys of adolescents, the PATH sample includes young people irrespective of school attendance, and its sampling frame includes college dormitories and children of active-duty military living in the US. More details about the PATH methodology are provided elsewhere [20]. The current study involved analysis of de-identified PATH Public Use data files only, and therefore is not considered human subject research. Data files were downloaded from https://www.icpsr.umich.edu/icpsrweb/NAHDAP/studies/36498 on Dec. 13, 2019 (waves 2, 3, and 4), Sep. 18, 2020 (wave 4.5), and October 13, 2021 (wave 5).

At wave 1, PATH recruited a “shadow sample” of youth 9 to 11 years of age to be interviewed in later waves once the youth became 12 years of age. Therefore, some individuals in the shadow sample may have been interviewed in wave 2, when they had turned 12 while others would have been interviewed in later waves of the study. In addition, a replenishment sample was drawn at wave 4 to compensate for attrition over time. Therefore, the PATH study can be considered a dynamic cohort. In order to fully utilize all available information across all waves, we structured the data such that the first data point corresponds to the first assessment of the individual (“Time 1”), the second data point corresponds to the second assessment (“Time 2”), and so on. As a result, a youth from the wave 2 cohort can contribute up to five data points across all waves with up to four person-intervals between assessments. Similarly, a youth in the ‘shadow sample’ who became eligible in wave 3 can have up to four data points, with up to three person-intervals). All youth who responded to at least two consecutive assessments were included. This approach allowed us to capture the dynamic nature of the cohort and maximize the statistical power of our analyses.

### Assessment

Audio computer assisted self-interviews (ACASI) with standardized multi-item modules were used to assess tobacco use history and a range of related variables. Current e-cigarette use was defined as using an e-cigarette product in the 30 days prior to the assessment. Peer tobacco use was assessed via the question “how many of your best friends use e-cigarettes?” Response options were “none,” “a few,” “some,” “most,” and “all.” Peer e-cigarette use was defined as having at least a few friends who used the tobacco product of interest. We coded “don’t know” and “refused” responses to missing. Once participants turned 18, they were administered the adult version of the questionnaire, which did not collect information about peer e-cigarette use. Therefore, our analytic sample included adolescents who were 12–17 during the study period.

Information about sex (male or female) and age categories (12–14 or 15–17 years of age at baseline, as provided in the PATH public use file) were extracted from survey items in the Demographics module. When these items were missing, information from the household screening roster was drawn.

### Analysis

First, we provided descriptive statistics (i.e., unweighted frequencies and weighted proportions) for peer e-cigarette use and e-cigarette use at each assessment.

**Fig. 1:**
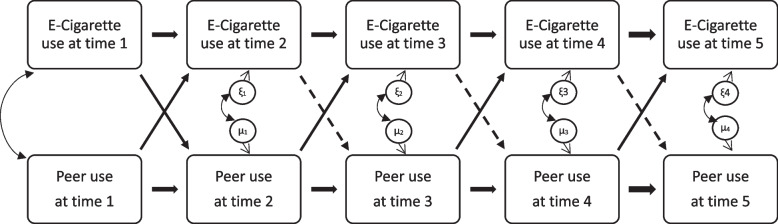
Results from cross-lagged model. Data from PATH youth surveys wave 2 to wave 5 (2014 to 2019). Solid lines represent paths that were statistically significant at 0.05 level. Dashed lines represent paths that were not statistically significant at 0.05 level. Observed variables are shown in rectangles and unobserved variables (error terms) are shown in circles. Similar variables are presented in rows, and variables from the same assessment wave are shown in columns. Curved lines represent covariances. Straight lines represent regression relationships. Note that we structured the data from multiple PATH waves such that the first data point corresponds to the first assessment of the individual (“Time 1”), the second data point corresponds to the second assessment of the individual (“Time 2”) and so on. As a result, a youth from the wave 2 cohort can contribute up to five data points across all waves, with up to four person-intervals between assessments. Similarly, a youth in the replenishment ('shadow sample') who became eligible in wave 3 can have up to four data points, with up to three person-intervals

To answer our research question, we used a cross-lagged model (see Fig. 1 for a depiction) to deconstruct the peer-own-tobacco-use relationships into (a) same variable predicting itself (autoregressive relationships), (b) peer or own tobacco use predicting each other in the future (cross-lagged relationships), and (c) correlations at the same assessment.

Using the aforementioned analytical approach, we gain insights about the nature of the relationship by stipulating specific elements of these relationships over time and provide insights toward the relative strengths of “peer-influence” pathways and “peer-selection” pathways while accounting for the liability of e-cigarette use via autoregressive paths in cross-lagged models. With this approach, we sought to provide insights towards a better understanding of the relationship between peer and own e-cigarette use.

We conducted all analyses for 12–17-year-olds as a whole as well as for subgroups stratified by age groups (i.e., 12–14 years and 15–17 years, as provided in the PATH public use file) and sex (i.e., boys and girls) to explore potential sex- and age-related variations. Potential subgroup variations were statistically assessed using product terms in GLM and testing restrictions on model parameters in multigroup analysis of cross-lagged models (using Model Test command in Mplus).

PATH-derived sample weights for the most recent observation were used to account for selection probabilities, possible deficiencies in the sampling frame, and missingness due to nonresponse patterns and attrition [20]. Variances of estimates were produced using balance repeat replication methods (Fay’s method with fay = 0.3). A robust weighted least square mean and variance (WLSMV) adjusted estimator, which uses a full weight matrix, was used to accommodate categorical variables and complex survey design in structural equation models. Analyses were conducted using Stata 16.0 (StataCorp, College Station, Texas, USA) and Mplus 8.1 (Muthén & Muthén, Los Angeles, CA, USA). Multiple fit indices were used to evaluate the goodness of fit of the cross-lagged model, including root mean square of approximation (RMSEA) [21], comparative fit index (CFI) [22], and Tucker-Lewis index (TLI). A RMSEA < 0.08 and CFI/TLI > 0.90 were considered as indications of reasonably good model fit [23, 24].

## Results

The analysis included 50,647 person-time data points aggregated from 13,074 adolescents. At baseline (time 1), the sample consisted of 48.6% (95% CI = 48.2% to 48.9%) of girls and 62.9% (95% CI = 62.3% to 63.5%) 12–14-year-olds; 53.2% (95% CI = 52.7% to 53.7%) were non-Hispanic White, 23.8% (95% CI = 23.4% to 24.2%) were Hispanics, 12.9% (95% CI = 12.6% to 13.2%) were non-Hispanic Black, and 10.1% (95% CI = 9.8% to 10.5%) were non-Hispanic Others. The prevalence of e-cigarette use increased from 1.9% at time one to 3.7%, 4.3%, 8.0%, and 12.1% at subsequent time points. As shown in Table 1, at each time point, 74.6% to 86.5% of adolescents who used e-cigarettes in the past 30 days had at least a few best friends who also used e-cigarettes. In contrast, a lower proportion (12.9% to 40.4%) of adolescents who did not use e-cigarettes in the past 30 days had at least a few best friends who used e-cigarettes.

**Table 1:** Prevalence of current peer e-cigarette use among adolescents 12–17 years of age. Data from PATH youth surveys wave 2 to wave 5 (2014–2019)

	Overall		Among current non-e-cigarette users		Among current e-cigarette users	
Peer e-cigarette use	n	% (95% CI)	n	% (95% CI)	n	% (95% CI)
All						
Time 1	12,875	15.2 (14.3, 16.1)	12,630	12.9 (13.1, 14.7)	245	81.6 (75.6, 86.4)
Time 2	12,784	21.2 (20.3, 22.2)	12,326	19.2 (18.4, 20.1)	458	74.6 (70.8, 78.0)
Time 3	10,427	21.5 (20.4, 22.6)	10,012	18.9 (17.9, 20.0)	415	79.1 (75.0, 82.6)
Time 4	8195	33.8 (32.4, 35.2)	7573	29.2 (27.9, 30.6)	622	86.5 (83.4, 89.2)
Time 5	6366	45.7 (44.2, 47.2)	5618	40.4 (38.7, 42.0)	748	84.3 (81.4, 86.8)
Males 12–14 years						
Time 1	4733	10.1 (9.3, 11.0)	4680	9.3 (8.5, 10.2)	53	73.9 (60.7, 83.8)
Time 2	4681	16.1 (14.9, 17.4)	4578	15.0 (13.7, 16.3)	103	63.1 (53.6, 71.7)
Time 3	4504	19.5 (18.2, 20.9)	4359	17.5 (16.2, 18.9)	145	76.2 (69.0, 82.1)
Time 4	4169	33.8 (32.1, 35.6)	3873	29.7 (27.9, 31.5)	296	84.1 (78.7, 88.3)
Time 5	3250	44.4 (42.4, 46.4)	2879	39.4 (37.4, 41.5)	371	81.5 (76.5, 85.6)
Males 15–17 years						
Time 1	1908	32.2 (29.4, 35.1)	1828	29.6 (27.1, 32.4)	66	86.6 (75.4, 92.7)
Time 2	1932	36.9 (34.5, 39.3)	1790	33.1 (30.8, 35.6)	142	80.7 (73.6, 86.2)
Time 3	865	37.5 (24.1, 41.0)	789	33.2 (30.0, 36.5)	76	78.2 (66.9, 86.4)
Time 4	52	52.9 (38.0)	43	43.7 (28.0, 60.7)	9	89.0 (47.0, 98.7)
Females 12–14 years						
Time 1	4430	9.6 (8.7, 10.7)	4392	9.0 (8.1, 10.1)	38	75.7 (61.0, 86.1)
Time 2	4354	16.3 (15.1, 17.6)	4269	14.9 (13.7, 16.2)	85	82.7 (72.9, 89.5)
Time 3	4205	18.5 (17.0, 20.2)	4086	16.6 (15.1, 18.2)	119	81.6 (74.0, 87.5)
Time 4	3899	33.6 (32.0, 35.4)	3596	28.7 (27.1, 30.3)	303	89.8 (85.7, 92.8)
Time 5	3090	47.2 (44.9, 49.5)	2717	41.7 (39.2, 44.2)	373	86.9 (83.4, 89.9)
Females 15–17 years						
Time 1	1771	27.6 (25.0, 30.4)	1698	25.0 (22.5, 27.6)	73	87.2 (73.9, 94.2)
Time 2	1781	32.9 (30.3, 35.5)	1654	29.7 (27.3, 32.2)	127	72.2 (62.7, 80.0)
Time 3	819	32.7 (29.5, 36.2)	745	27.2 (24.2, 30.5)	74	81.3 (29.5, 36.2)
Time 4	46	44.9 (29.9, 60.9)	34	33.4 (18.8, 52.0)	12	75.5 (40.7, 93.2)

Our analyses include data from five time points: waves 2, 3, 4, 4.5, and 5. In order to fully utilize all available information across all waves, we structured the data such that the first data point (“Time 1”) corresponds to the first assessment of the individual, the second data point corresponds to the second assessment (“Time 2”), and so on. As a result, a youth from the wave 2 cohort can contribute up to five data points across all waves, with up to four person-intervals between assessments. Similarly, a youth in the replenishment ('shadow sample') who became eligible in wave 3 can have up to four data points, with up to three person-intervals

The cross-lagged model as shown in Fig. 1 fit the data reasonably well based on fit indices (CFI = 0.994; TLI = 0.986; RMSEA = 0.014, 90% CI = 0.010 to 0.019). Table 2 shows standardized regression coefficients of the cross-lagged model of the overall sample as well as sex- and age-subsamples**,** and Fig. 1 visualized results of the overall sample. All autoregressive paths were highly significant, signaling strong prediction of the same behavior (i.e., own tobacco use and peer tobacco use) from one time point to the next. With respect to cross-lagged paths, peer e-cigarette use consistently predicted own e-cigarette use at the next time point, whereas own e-cigarette use did not predict peer e-cigarette use at the next time point except for from time 1 to time 2. The covariance term at time 1 and correlations of error terms at other time points were highly significant (p < 0.001). Consistent with the observation that estimates were largely similar for girls and boys, results from the Wald test of parameter constraints indicated that all prediction paths were equal between boys and girls (p = 0.067). For age subgroups, we were only able to test the equality of paths from time 1 to time 3 because 15–17-year-olds had become adults by time 4. The prediction paths were equal between 12–14-year-olds vs. 15–17-year-olds (p = 0.064). To better control for potential cohort effect (e.g., in relation to changes in the availability, marketing practices, and perceptions about e-cigarette products), we conducted analysis using only the wave 2 cohort. Results and statistical inferences were similar and are available upon request.

**Table 2:** Path estimates (standardized) from cross-lagged models for own and peer e-cigarette use. Data from PATH youth surveys wave 2 to wave 5 (2014 to 2019)

		**All**		**Early adolescents (12–14-year-olds)**				**Late adolescents (15–17-year-olds)**			
		**n = 13,074**		**Female (n = 4488)**		**Male (n = 4814)**		**Female (n = 1792)**		**Male (n = 1942)**	
	Time	β	95% CI	β	95% CI	β	95% CI	β	95% CI	β	95% CI
**Autoregressive paths**											
Own e-cigarette use	1 to 2	**0.16**	**0.14 to 0.19**	**0.14**	**0.07, 0.20**	**0.15**	**0.10, 0.19**	**0.18**	**0.11, 0.25**	**0.20**	**0.14, 0.26**
	2 to 3	**0.59**	**0.51 to 0.68**	**0.45**	**0.21, 0.69**	**0.65**	**0.53, 0.77**	**0.54**	**0.35, 0.73**	**0.51**	**0.31, 0.72**
	3 to 4	**0.71**	**0.63 to 0.79**	**0.67**	**0.56, 0.79**	**0.69**	**0.59, 0.80**				
	4 to 5	**0.71**	**0.62 to 0.80**	**0.67**	**0.52, 0.82**	**0.72**	**0.60, 0.84**				
Peer e-cigarette use	1 to 2	**0.48**	**0.46 to 0.50**	**0.46**	**0.42, 0.49**	**0.43**	**0.40, 0.46**	**0.45**	**0.40, 0.50**	**0.46**	**0.42, 0.51**
	2 to 3	**0.66**	**0.60 to 0.72**	**0.79**	**0.57, 1.01**	**0.70**	**0.61, 0.79**	**0.42**	**0.27, 0.57**	**0.61**	**0.45, 0.78**
	3 to 4	**0.61**	**0.48 to 0.74**	**0.48**	**0.31, 0.64**	**0.64**	**0.51, 0.76**				
	4 to 5	**0.69**	**0.62 to 0.77**	**0.69**	**0.56, 0.82**	**0.70**	**0.59, 0.81**				
**Prediction paths**											
Peer e-cigarette use predicting own e-cigarette use	1 to 2	**0.29**	**0.26 to 0.32**	**0.22**	**0.16, 0.28**	**0.23**	**0.18, 0.29**	**0.31**	**0.22, 0.39**	**0.29**	**0.22, 0.35**
	2 to 3	**0.21**	**0.13 to 0.29**	**0.34**	**0.13, 0.55**	**0.17**	**0.06, 0.29**	0.19	-0.01, 0.38	**0.20**	**0.03, 0.38**
	3 to 4	**0.12**	**0.03 to 0.21**	**0.15**	**0.03, 0.27**	**0.13**	**0.01, 0.25**				
	4 to 5	**0.10**	**0.01 to 0.19**	0.13	-0.03, 0.29	0.11	-0.03, 0.24				
Own e-cigarette use predicting peer e-cigarette use	1 to 2	**0.06**	**0.03 to 0.09**	**0.06**	**0.01, 0.10**	0.04	-0.00, 0.09	0.04	-0.03, 0.10	**0.14**	**0.09, 0.19**
	2 to 3	0.08	-0.01 to 0.16	-0.16	-0.45, 0.13	0.02	-0.14, 0.18	**0.38**	**0.20, 0.56**	0.03	-0.20, 0.27
	3 to 4	0.15	-0.04 to 0.33	**0.34**	**0.13, 0.55**	0.08	-0.11, 0.26				
	4 to 5	-0.03	-0.15 to 0.09	0.01	-0.17, 0.18	-0.06	-0.23, 0.11				

Bold font indicates statistical significance at 0.05 level

## Discussion

This study examines the association between peer e-cigarette use and own e-cigarette use among U.S. adolescents using data from waves 2–5 of the Population Assessment of Tobacco and Health (PATH) study. Despite the growing concern about adolescent e-cigarette use and the potential influence of peers, few studies have explored the longitudinal mutual relationships between peer and own e-cigarette use. This study fills this gap by examining the extent to which peer e-cigarette use predicts own use over time.

Our study expands on previous evidence of strong associations between peer and own tobacco use among adolescents by examining the mutual relationships over time in the context of e-cigarette use. Our results support the role of peer influence rather than peer selection in e-cigarette use among youth, as demonstrated by our use of a cross-lagged model. (The statistically significant path from own to peer e-cigarette use between time 1 and 2 is likely a result of a greater statistical power from larger sample sizes at earlier time points. At later time points, sample sizes became smaller due to participants aging out (i.e., becoming adults) and attrition (as shown in Table 1). Nonetheless, the strength of prediction was much weaker compared to the peer-to-own-use paths (i.e., 0.06 vs. 0.29).)

Our study reveals a key difference between the peer influence process for e-cigarette use among adolescents and the peer selection process observed in cigarette smoking among youth (i.e., seeking out friends who also smoke) [16–19]. Although both behaviors are influenced by social factors such as peer use, e-cigarette use may be more susceptible to peer influence compared to cigarette smoking, potentially due to differences in social norms and perceptions. For example, during the study period (i.e., 2014–2019), e-cigarette use became the most common tobacco product used while cigarette smoking continued to decline [25, 26]. It is possible that e-cigarettes are more socially accepted and perceived as fashionable compared to cigarettes among adolescents [27]. As such, use of e-cigarettes among one’s peers can have a greater influence on one’s own decision to use the products. As a relatively new type of product, e-cigarettes may appeal to adolescents’ novelty seeking tendency [28]. Further, e-cigarettes may be perceived as less harmful or less socially stigmatized compared to cigarette smoking, which could influence the degree to which peers influence each other's behavior. These differences in perception about e-cigarette use and cigarette smoking may contribute to the difference in peer influence vs. peer selection processes to the extent that smoking may be less socially accepted, and individuals who smoke may be more motivated to seek others who smoke. Supportive evidence includes findings that some adolescents perceived e-cigarettes as “cool” or used as a vehicle to socialize with peers [29, 30]. Further research is needed to fully understand the nuances of how peer influence operates in the context of e-cigarette use and how it may differ from traditional cigarette smoking. It is an important area of study given that the prevalence of underage e-cigarette use remains a significant public health concern. Future research comparing reasons to use between e-cigarettes and cigarettes will further be able to shed new light on mechanisms underlying these observed differences.

Findings from this study have important public health implications – peers may be key players in preventing youth e-cigarette use. For example, prevention programs may benefit from incorporating peer-oriented strategies such as fostering resistance to peer pressure among nonusers or using peer-lead approaches to reduce e-cigarette use among users. In a recent publication, Chu and colleagues showed the feasibility of such a program: peer-lead approaches were well accepted among teachers and students [31, 32]. Compared to an expert-led approach, peer-led approaches were more engaging; despite being statistically non-significant partially due to the limited sample size, point estimates suggested that peer-led approaches may be more effective than an expert-lead approach [31]. Previous studies on adolescent alcohol use further suggest that peer influence can be a malleable socio-environmental factor for prevention and intervention [33]. Expanding such research to e-cigarette use initiation and behaviors may shed new light on whether peers can be potential venues for youth tobacco prevention and intervention.

In contrast to previous findings about age- and sex-related variations in the relationship between peer and own e-cigarette use [14] and cigarette smoking [6, 13], we found limited evidence for differences by age and sex. A few methodological differences may contribute to the divergence, including the study design (longitudinal vs. cross-sectional), study population (national vs. regional samples), study period, or statistical models used. It is noteworthy that we were not able to fully investigate the relationship among older adolescents using the cross-lagged model as they became adults in later time points. Nonetheless, our findings provide insights for future studies and are in line with previous studies showing that peer use remains an important factor for e-cigarette use throughout adolescence.

Findings from this study should be interpreted with the following limitations in mind. First, the study is observational in nature. Although the cross-lagged models took into account previous behaviors, potential effects of time-varying variables could not be ruled out. Evidence from experimental studies will complement findings from this study to gauge the size of the potential effect of peer influence on e-cigarette use. Mechanistic research that incorporates potential mediators, such as perceived benefits and self-efficacy, can bear important implications for intervention [33]. Second, we focused on current use status in this study and provided overall estimates. Future studies that separate the onset and persistence of use processes will be able to assess any potential variation by stages of e-cigarette use. In a previous study, we found that best friends offering predicted the onset of e-cigarette use among youth [34]. Another study using PATH data found that having friends who used e-cigarettes predicted both initiation and persistence of use; stronger association was found at the initiation stage [7]. A study on different stages of e-cigarette use will provide further insights about the nature of the relationship. Third, all information was based on self-reporting, which is of reasonable validity [35]. Nonetheless, studies that incorporate bio-verification are less prone to misclassification. In addition, information about peer e-cigarette use was not available once participants turned 18, which, along with attrition, resulted in lower statistical powers at later time points. Fourth, the prevalence of e-cigarette was relatively low at time 1 in this sample as a result of participants being younger and e-cigarettes being a relatively new product at earlier waves of the study. The associations between peer and own e-cigarette use might change as e-cigarette become more common in the marketplace, especially for older youth who had become adults in later waves. Results based on more recent data will shed important light on this issue. Finally, we explored subgroup variations by sex and age, arguably two of the most important covariates in youth e-cigarette use, in this study. Future studies on potential subgroup variations by other characteristics may provide useful information for tailored prevention and intervention strategies. Counterbalancing strengths of the study include (a) the use of a nationally representative sample facilitated the generalization of results to household dwelling youths in the US; and (b) the longitudinal design with five waves of data enabled the use of the cross-lagged model to further interrogate mutual relationships with clear temporal sequences.

## Conclusions

We found that peer influence paths were more robust than peer selection paths for youth e-cigarette use.

### Supplementary Information

**Additional file 1.** 

## Data Availability

The datasets generated and/or analyzed during the current study are available at https://www.icpsr.umich.edu/icpsrweb/NAHDAP/studies/36498.

## Abbreviations

US: United States
NYTS: National Youth Tobacco Survey
GLM: Generalized linear regression
PATH: Population Assessment of Tobacco and Health
ACASI: Audio computer assisted self-interviews
WLSMV: Weighted least square mean and variance
RMSEA: Root mean square of approximation
CFI: Comparative fit index
TLI: Tucker-Lewis index
