# Supplementary material for: Mutual pathways between peer and own e-cigarette use among youth in the United States: a cross-lagged model
Source: BMC Public Health. 2023 Aug 24;23:1609. doi: 10.1186/s12889-023-16470-5 (PMC10463603; doi:10.1186/s12889-023-16470-5)
Supplement: Supplementary file 1 — Additional file 1. [file 12889_2023_16470_MOESM1_ESM.docx]

**Supplemental Material**

1. **Definition of covariates**

**Race/ethnicity** was categorized into non-Hispanic white, Hispanic, non-Hispanic black, and non-Hispanic others based on derived variables provided in the PATH Public Use File.

**School performance** was dichotomized into “mostly A’s and B’s” and “others” based on parents’ responses to the question “How would you describe how {Child's first name} has performed at school in the past 12 months? Would you say {Child's first name}'s grades are...?”

**Household tobacco use** (coded as ‘yes’ or ‘no’) was based on the responses to the question “Do you think cigarettes or tobacco might be available to {Child's first name} at your home?” from the adolescent’s parent or legal guardian.

**Past 30-day alcohol drinking** (coded as ‘yes’ or ‘no’) was based on the adolescent’s response to the question “Have you used alcohol in the past 30 days?”

**Past 30-day cannabis use** (coded as ‘yes’ or ‘no’) was based on the adolescent’s response to the question “Have you used marijuana in the past 30 days?” In addition, adolescents who indicated that they used blunt (i.e., “cigar, cigarillo, or filtered cigars with marijuana in it”) in the past 30 days were also coded as “yes” in this variable.

**Perceived harm of e-cigarette use** was dichotomized into “perceiving a lot of harm” and “not perceiving a lot of harm” based on the adolescent’s response to the question “How much do you think people harm themselves when they use e-cigarettes?”

“Don’t know” and “refused” were coded as missing for all above variables.
